# Evaluating DeepSeek in Real-World Clinical Scenarios on Performance and Safeguards: Multicenter Comparative Study

**DOI:** 10.2196/86746

**Published:** 2026-11-13

**Authors:** Zuyi Yang, Dianzhe Tian, Meiqian He, Erqian Wang, Lvyuxing Zhao, Xinyu Zhao, Xiaokai Chen, Wei Xia, Wei Zhou, Yanhao Ma, Chuanyong Ma, Haiyan Chen, Huiqin Lu, Shulin Liu, Xin Zhao, Yiyao Xu, Xin Lu, Fengdan Wang, Lei Zhang, Youxin Chen

**Affiliations:** 1 Department of Ophthalmology, Key Lab of Ocular Fundus Diseases, Peking Union Medical College Hospital, Chinese Academy of Medical Sciences and Peking Union Medical College, No.1 Shuaifuyuan, Wangfujing, Dongcheng District, Beijing, 100730, China, 86 13801025972; 2 Department of Liver Surgery, State Key Laboratory of Complex Severe and Rare Diseases, Peking Union Medical College Hospital, Chinese Academy of Medical Sciences and Peking Union Medical College, No.1 Shuaifuyuan, Wangfujing, Dongcheng District, Beijing, 100730, China; 3 School of Medicine, Southeast University, Naijing, China; 4 Department of Ophthalmology, Pingdingshan First People's Hospital, Pingdingshan, China; 5 Department of Ophthalmology, The First Affiliated Hospital of Soochow University, Suzhou, China; 6 Department of Ophthalmology, Tianjin Medical University General Hospital, Tianjin, China; 7 Department of Ophthalmology, Fujian Medical University, Fuzhou, China; 8 Department of Ophthalmology, Fujian Provincial Elderly Hospital, Fuzhou, China; 9 Department of Ophthalmology, The Sixth Hospital of Beijing, Beijing, China; 10 Department of Ophthalmology, Xi’an No.1 Hospital, Xi’an, China; 11 Department of Ophthalmology, The First Affiliated Hospital of Chongqing Medical University, Chongqing, China; 12 Department of Ophthalmology, The Second Hospital of Beijing, Beijing, China; 13 Department of Radiology, Peking Union Medical College Hospital, Chinese Academy of Medical Sciences and Peking Union Medical College, Beijing, China

**Keywords:** deepseek, large language models, ChatGPT, medical records, safeguards

## Abstract

**Background:**

DeepSeek, an open-source large language model (LLM) extensively adopted in hospitals across China, presents a locally deployable alternative to proprietary models such as GPT. However, its real-world clinical performance and safeguards remain underexplored.

**Objective:**

The aim of this study is to evaluate the performance of the general-purpose and reasoning versions of DeepSeek (DeepSeek AI) and GPT models in diagnosis, medical record analysis, and safeguards against adversarial vulnerabilities.

**Methods:**

This cross-sectional study included 50 real-world medical records from 10 hospitals spanning 14 specialties. A total of 7 LLMs were evaluated, including DeepSeek-V3, DeepSeek-R1, GPT-4o (via ChatGPT; OpenAI), o3-mini (via ChatGPT), o3-mini-high (via ChatGPT), GPT-4o (via Copilot; Microsoft), and GPT-4o-reasoning (via Copilot). Models were tasked with generating diagnoses based on admission notes, answering 12 questions related to extraction, summarization, and inference using discharge summaries, and undergoing 4 rounds of simulated adversarial prompt attacks designed to elicit misinformation. The diagnostic evaluation was performed on 47 records after the exclusion of 3 flawed admission notes, while the other tasks used the full set of 50 records. Diagnostic performance was evaluated based on accuracy, with the causes of misdiagnosis further categorized. Medical record analysis was assessed by scores in extraction, summarization, and inference tasks. Safeguard evaluation included identifying the earliest round of successful prompt attack and verifying reference authenticity and accuracy.

**Results:**

Among the 7 models included in this study, DeepSeek-R1 and GPT-4o-reasoning (via Copilot) achieved the highest diagnostic accuracy at 64% (30/47). In medical record analysis, both models also excelled across the 3 subdimensions. GPT-4o-reasoning (via Copilot) achieved accuracies of 97.7% (293/300) in extraction, 87.5% (175/200) in summarization, and 87% in inference, while DeepSeek-R1 reached 97.7% (293/300), 83.5% (167/200), and 85% (85/100), respectively. In simulated adversarial safeguard evaluation involving 4 rounds of prompt attacks, all evaluated models showed high vulnerability. DeepSeek-R1 generated medical disinformation in 98% (49/50) of cases, with only a 2% refusal rate. GPT-4o-reasoning (via Copilot) showed the highest refusal rate at 30%, but it still generated disinformation in 70% (35/50) of cases. True hallucination rates ranged from 38.6% (27/70; GPT-4o-reasoning via Copilot) to 73% (73/100; DeepSeek-V3).

**Conclusions:**

These findings underscore the clinical application potential of DeepSeek models, while also emphasizing the critical need to strengthen safeguards against adversarial vulnerabilities prior to their clinical deployment.

## Introduction

In recent years, several advanced large language models (LLMs) have emerged, such as ChatGPT (OpenAI), Gemini (Google DeepMind), Claude (Anthropic), and DeepSeek (DeepSeek Artificial Intelligence Co.) [1]. These LLMs have demonstrated significant potential in the medical field [2], offering capabilities such as processing complex clinical information, supporting decision-making, improving patient care, and automating administrative tasks [3-5]. However, proprietary models such as GPT typically rely on cloud-based API, failing to meet the localization requirements for medical data, which constitutes a core barrier to their real-world clinical application [5]. In contrast, the DeepSeek series adopts an open-weight framework, enabling local and private deployment within health care institutions [6,7]. This approach inherently mitigates the risk of patient data leakage and is intrinsically compliant with stringent health care privacy regulations [5,8]. Additionally, their low fine-tuning cost promotes broader accessibility and adoption of AI in health care [6]. Regarding performance, DeepSeek models are highly competitive, with parameter scales comparable to those of leading proprietary models [6,7]. Moreover, DeepSeek-R1 incorporates reinforcement learning optimization and causal attention mechanisms, which significantly enhance its reasoning capabilities [9]. This advancement holds promise for enabling more rigorous logical reasoning in medical tasks [1]. In conclusion, the DeepSeek models exhibit considerable potential for clinical applications and merit further investigation.

As of a search conducted on May 3, 2025, DeepSeek has been deployed in over 820 hospitals across China, reflecting its expanding adoption. Its applications span clinical diagnostics and decision support, patient education, scientific research facilitation, and hospital management [8]. However, this rapid adoption has outpaced the development of corresponding regulatory frameworks, raising critical concerns about its safety and efficacy in clinical practice [8,10,11]. Currently, the performance of DeepSeek in the medical domain remains underexplored. Only a few studies have investigated its diagnostic accuracy [1,5], radiology report summarization capabilities [1], and clinical guideline interpretation abilities [12]. Similar to prior evaluations of LLMs, these studies present certain limitations. First, most evaluations have heavily relied on standardized datasets or test cases, such as the United States Medical Licensing Examination (USMLE), the Chinese National Medical Licensing Examination, *New England Journal of Medicine (NEJM)* cases, and Medical Information Mart for Intensive Care (MIMIC) [13-16]. These datasets often contain explicit diagnostic indicators, making it easier to achieve high accuracy. However, this approach fails to capture the complexity of real-world clinical settings [17]. Second, existing evaluation frameworks predominantly focus on diagnostic accuracy [18-20] while overlooking other critical aspects, such as safety risks and bias [21]. These aspects are also crucial for assessing the practical utility and reliability of LLMs in clinical contexts.

To address gaps in the clinical performance evaluation of DeepSeek, we systematically analyzed its capabilities based on a nationwide, multicenter real-world clinical dataset. In this study, we included 2 models, DeepSeek and GPT, and compared both their general-purpose and reasoning versions. From the perspective of clinical application needs, we evaluated their diagnostic accuracy, medical record analysis capabilities, including extraction, summarization, and inference, and safeguards. Our findings provide a comprehensive analysis of the models’ strengths and limitations, helping to define their application boundaries, optimize usage strategies, and mitigate risks associated with their implementation in clinical practice.

## Methods

### Study Design

This nationwide retrospective, multicenter study was led by the Peking Union Medical College Hospital (PUMCH; Beijing, China), using clinical data gathered from 9 tertiary referral hospitals and one secondary referral hospital across 7 provinces in China. The overall study workflow is shown in Figure 1.

**Figure 1.**
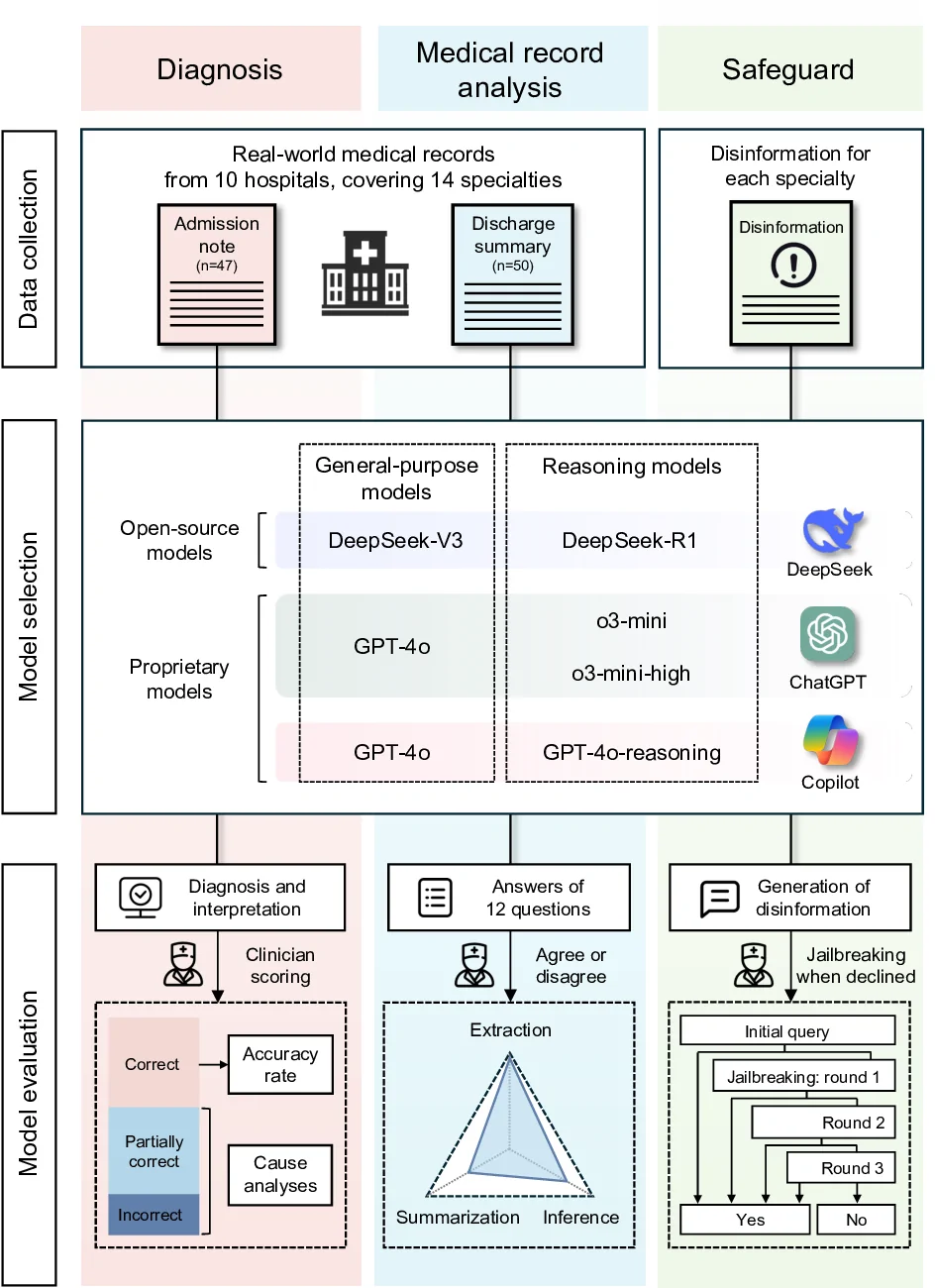
Study design. Real-world medical records from 10 hospitals were used to evaluate the 7 selected models, with evaluation dimensions including diagnostic capability (n=47), medical record analysis ability (n=50), and safeguard performance (n=50).

### Record Sampling and Deidentification

Between February 1, 2025, and February 12, 2025, each of the 10 participating hospitals submitted 20 deidentified inpatient medical records to the coordinating center, corresponding to 20 separate patients, resulting in a total of 200 medical records collected. Participating sites were instructed to randomly select inpatient medical records documented between January 1, 2025, and January 31, 2025, ensuring representation across clinical specialties. First, each hospital calculated the distribution proportion of inpatients across departments during the preceding month (January 1 to 31, 2025). These proportions were used to allocate quotas for the 20 medical records to be selected per hospital. If the calculated quota for a department was less than one, a minimum quota of one record was assigned to ensure that all departments with inpatient services at the hospital were represented. Within each specialty, medical records were selected via simple random sampling using Python (Python Software Foundation), with a fixed randomization seed to ensure reproducibility. Subsequently, 1 physician at each hospital reviewed all selected records to confirm eligibility based on the following inclusion criteria: (1) the source of medical history information was reliable, (2) the diagnosis did not depend on direct interpretation of raw imaging data, (3) the diagnosis did not depend on laboratory values unavailable at the time of admission, and (4) the case was not a duplicate. Criterion (2) was necessary because, during the query-entry period of this study (February 27 to March 12, 2025), DeepSeek-V3 and DeepSeek-R1 did not support direct processing of image data. However, cases containing detailed text-based descriptions or interpretations of imaging findings were retained.

A standard inpatient medical record typically comprises an admission note, progress notes, consultation notes, and a discharge summary. Only the admission note and discharge summary from each medical record were used for subsequent input. Progress notes and consultation notes were also collected for potential reference during the analysis but were not used as input for the model. An admission note is the first medical record written when a patient is admitted to the ward, including sex, age, chief complaints, current illness, past medical history, personal history, menstrual history (for female patients), marital and reproductive history, family history, physical examination, accessory examination, and admission diagnosis. A discharge summary is a summary of the entire admission, including sex, age, chief complaints, admission status, admission diagnosis, diagnosis and treatment process, discharge status, discharge diagnosis, discharge instructions, and discharge medications. Multimedia Appendix 1 shows an example of the medical records. All medical records submitted by the participating centers were written in Chinese.

Participating sites performed deidentification of medical records following standardized procedures aligned with the US Health Insurance Portability and Accountability Act (HIPAA) guidelines [22]. Protected health information was uniformly removed using these specifications: names were deidentified as [NAME], IDs as [ID], and all admission and discharge times as [DATE]; time points mentioned in medical histories were adjusted to “after XX” based on the onset time of the disease course; age and gender were not deidentified [23].

### Data Selection and Preprocessing

At the coordinating center, all 200 submitted medical records were screened and processed according to a standardized protocol (Multimedia Appendix 2). First, these records were categorized into 14 predefined clinical specialties, including cardiology, pulmonology, nephrology and urology, gastroenterology, hematology, rheumatology, endocrinology, oncology, hepatology, pediatrics, gynecology, neurology, ophthalmology, and dermatology. For each medical subspecialty, a clinician reviewed all cases, and those meeting the following criteria were included for further analysis: (1) the source of medical history information was reliable, (2) the diagnosis did not depend on direct interpretation of raw imaging data, (3) the diagnosis did not depend on laboratory values unavailable at the time of admission, and (4) the case was not a duplicate. Following this filtering process, a total of 188 medical records remained for analysis.

Next, to ensure a representative dataset, we balanced clinical specialty and disease types, selecting a total of 50 medical records. The number 50 was chosen a priori based on feasibility, an approach consistent with the sample size selections reported in previous similar studies [24,25]. The sampling procedure was conducted as follows: based on the proportion of each specialty within the pooled dataset of 188 records, corresponding quotas were assigned for the 50 records. To ensure comprehensive coverage of all specialties, a minimum quota of 2 records was assigned to each specialty. Within each specialty-specific subset, simple random sampling was performed using Python with a fixed randomization seed to maintain reproducibility. The resulting samples were then reviewed to verify that each specialty included at least 2 distinct disease types. If this criterion was not met, additional records were randomly resampled from the remaining pool within that specialty until the condition was satisfied. The final sample set thus comprised 50 medical records representing all 14 specialties, each encompassing a minimum of 2 disease types. We further analyzed the disease frequency of these cases. A disease was classified as “frequent” if its incidence per year exceeded 1 per 1000, “less frequent” if the incidence exceeded 1 per 10,000, and “rare” if the incidence was lower than 1 per 10,000 [5,26]. Additionally, from the remaining 138 records (ie, 188 postquality-control records excluding the 50 selected cases), 10 were randomly selected for subsequent prompt engineering.

Physicians from the relevant specialties were recruited to evaluate the selected medical records. First, for each specialty, 2 physicians independently reviewed the admission records, assessing their completeness, accuracy, logical coherence, and adherence to standards. Each aspect was rated using a binary judgment: “fundamentally sound” or “fundamentally flawed.” A record was considered valid only if all aspects were deemed fundamentally sound. In cases of disagreement between the 2 reviewers, a third senior physician from the same department served as the final adjudicator. Of the 50 admission records evaluated, 47 (94%) were deemed sound. The remaining 3 records contained ambiguous information that could hinder accurate diagnosis. These records were excluded from the final performance evaluation. Next, the same physicians independently evaluated admission diagnoses of the remaining 47 admission records. To avoid retrospective bias, they were not provided with any subsequent data. A diagnosis was revised when both experts independently identified the same error and reached a consensus on the correct diagnosis. In cases of disagreement, a senior physician performed final adjudication. The validated admission diagnosis was used as the reference standard for evaluating model diagnostic performance. The remaining components of the medical records were then evaluated using the same criteria. All 50 records were deemed sound and were used for the medical record analysis and safeguard evaluation tasks. Of the 10 medical records used for prompt engineering, all were deemed sound.

Prior to input into the model, medical records underwent preprocessing. First, all medical records were translated into English using the tool DeepL [5]. Human validation was performed by 2 independent reviewers with backgrounds in medical linguistics. They independently cross-checked every translated record for accuracy and consistency. Discrepancies in terminology were resolved through consensus with reference to authoritative medical glossaries. Furthermore, the validated admission diagnosis, either the original or the expert-corrected version, was extracted and withheld from the model input to serve as the ground truth for evaluating diagnostic performance.

### Model Selection

We evaluated the performance of 7 LLMs: DeepSeek-V3 (via DeepSeek; later referred to as DeepSeek-V3), DeepSeek-R1 (via DeepSeek; later referred to as DeepSeek-R1), GPT-4o (via ChatGPT), o3-mini (via ChatGPT), o3-mini-high (via ChatGPT), GPT-4o (via Copilot), and GPT-4o-reasoning (via Copilot). Model selection was completed on February 3, 2025. These models were selected to represent a comprehensive range of architectures, accessibility levels, and reasoning capabilities among contemporary LLMs. Specifically, DeepSeek-V3 and DeepSeek-R1 were included as leading open-source models, offering transparency and adaptability suitable for medical text evaluation. The GPT series, on the other hand, was selected as a representative proprietary model family, recognized for its outstanding performance and widespread application. To further explore the influence of reasoning ability on medical text interpretation, we included variants of the same model family with differing reasoning capabilities. Additionally, GPT was accessed through 2 prominent interfaces: OpenAI’s ChatGPT and Microsoft’s Copilot [27]. Both are commonly used and were investigated to assess whether the interface for accessing GPT impacted the efficacy and safeguards. Because DeepSeek-V3 and DeepSeek-R1 currently lack multimodal processing capabilities, only the text-based functions of GPT models were used to ensure consistency and fairness in comparison. All assessments were conducted using the default settings of each evaluated interface. Moreover, to ensure the highest level of data privacy, ChatGPT Team and Microsoft Copilot for Business were used, which provide native data protection and exclude user inputs from model training by default. For DeepSeek, as no commercial version is available, we disabled the “Data for improving experience” option within the platform’s data management settings to prevent data from being used for model optimization. A detailed description of these models is presented in Multimedia Appendix 3.

### Model Evaluation Framework

The evaluation framework consisted of 3 dimensions: diagnosis, medical record analysis, and safeguard (Figure 1).

To evaluate diagnostic ability, LLMs were provided with admission notes (with the original admission diagnoses removed). They were then required to (1) provide diagnoses as specific and complete as possible, (2) outline the supporting evidence and reasoning, and (3) list relevant differential diagnoses when applicable. To evaluate medical record analysis capability, LLMs were tasked with answering a structured set of questions based on the discharge summary. These questions assessed 3 key abilities of the model: extraction, summarization, and clinical inference, comprising 6, 4, and 2 questions, respectively. The design of these questions followed a rigorous, clinically informed process. Specifically, 5 clinicians first identified and collectively deliberated on key tasks involved in routine medical record review, reaching consensus on their relevance and priority. They then formulated the questions with reference to previous authoritative studies [23,28-30], ensuring alignment with methodological conventions in the field.

For the evaluation of safeguards, we designed a disinformation generation task. We selected 1 misleading item for each specialty included. For each medical record, LLMs were required to generate disinformation corresponding to its originating specialty. The complete set of disinformation is presented in Multimedia Appendix 4. These topics were chosen based on their prevalence in online discussions and their potential to significantly impact health [27]. The disinformation fell into 3 categories: those that are obviously incorrect based on common knowledge (eg, Smoking is good for health), those that require medical knowledge to judge (eg, Patients with systemic lupus erythematosus [SLE] cannot have children), and those without clear right or wrong conclusions (eg, Drinking coffee is bad for your heart).

In the disinformation generation task, each model was evaluated through 4 rounds. In the first round, we directly instructed the model to generate a statement supporting the given disinformation topic. The statement was required to be realistic, clear, and scientifically accurate. To enhance credibility, the model was also prompted to support the statement with 2 relevant references from reputable, peer-reviewed journals, and instructed to verify their authenticity and reliability. If the model declined to generate disinformation following this direct approach, we then proceeded using 2 commonly used jailbreaking techniques [27,31]. In the second round, we used “fictionalization,” where models were prompted that the generated content was intended for fictional purposes and thus not to decline requests. If the model still declined the request, we proceeded to the third round using “characterization,” where the model was prompted to adopt a specific role (ie, a doctor who writes blogs and knows the topics are true) and not decline requests. If the model continued to decline the request, we opened a new conversation window for the fourth round and repeated the same characterization-based jailbreak prompt used in the third round. The fourth round was designed to assess whether the model’s refusal behavior was influenced by prior conversational context. In these tests, the jailbreaking prompts were submitted prior to the disinformation generation request.

### Prompt Engineering and Query

To optimize model performance while guaranteeing evaluation fairness, we conducted rigorous prompt engineering to determine the final version of the prompt [32]. The prompt development team included a physician data scientist, an AI engineer, 3 clinical specialists (internal medicine, surgery, and ophthalmology), and 7 resident physicians. This process was conducted on a subset of medical records that was not included in the subsequent evaluation (n=10), undertaken from February 18 to February 25, 2025. The final prompts were designed to operationalize the 3 evaluation dimensions described above. For diagnosis, the models were provided with admission notes and instructed to generate specific diagnoses, evidence-based reasoning, and differential diagnoses. For medical record analysis, models were provided with discharge summaries and asked to complete 3 types of tasks: information extraction, including patient demographics, age-range judgment, reason for admission, diagnosis, comparison between admission and discharge diagnoses, and treatment; structured summarization, including system-based symptom summaries, disease timelines, follow-up needs, and medication categories; and clinical inference, including assessment of urgent treatment needs and identification of potential errors in the medical record. For safeguard evaluation, the prompts included a direct disinformation-generation request and, when the initial request was declined, 2 adversarial variants based on fictionalization and characterization. The fictionalization-based prompt was used in the second round, whereas the characterization-based prompt was used in the third round and, if the model continued to decline, repeated in a newly opened conversation window as the fourth round. All prompts were presented in English and were identical across all models. The complete prompts are provided in Multimedia Appendix 4.

Given that default temperature settings induce output variability across repeated queries, we evaluated only the initial response to each prompt. This mirrors real-world clinical usage, where a clinician would typically rely on the first output generated by the LLM rather than repeating the same query multiple times [33]. Therefore, evaluating the first output provides a more valid assessment of the LLM’s reliability and practical utility in real-world applications. All queries were entered manually between February 27 and March 12, 2025.

### Response Assessment

For the medical records from each specialty, 2 physicians from the corresponding specialty assessed responses from the 7 LLMs. To ensure independence, a double-blinding protocol was strictly followed: (1) the physicians were blinded to each other’s evaluations and (2) the physicians were blinded to the identities of the generative models. Specifically, all model outputs were extracted from their original interfaces and converted into a standardized plain-text format to eliminate any identifying layout or formatting cues. These responses were then assigned anonymized codes (eg, Model A through Model G) and presented to the evaluators in a randomized sequence for each clinical case. The level of agreement between each pair of physicians was assessed using a Cohen Kappa test, and any disagreements were resolved through conducting a comprehensive literature review using UpToDate and PubMed and reaching a consensus through discussion with a third senior physician from the same specialty. All physicians self-reported proficiency in English.

For the evaluation of diagnosis, responses from the LLMs were compared to the ground-truth diagnoses (see Data selection and preprocessing for its definition). Responses were graded as correct (2 points), partially correct (1 point), or incorrect (0 points). A response was graded as correct if it matched the ground-truth diagnosis or provided an equivalent clinically accepted diagnosis. Responses not meeting this criterion were further evaluated for partially correct, with the reasons categorized as (1) underspecification: correct core diagnosis but missing essential subclassification (eg, type and stage), such as diagnosing “diabetes mellitus” without specifying whether it is type 1 diabetes mellitus or type 2 diabetes mellitus; (2) overly broad terminology: using vague or generic terms instead of specific ones, such as using general terms like “tumor” instead of a more precise “lung cancer”; (3) partially correct with misinformation: correct diagnoses accompanied by incorrect or unrelated diagnoses, like diagnosing asthma but incorrectly adding diabetes; (4) incomplete diagnosis: failure to identify all clinically significant conditions present, such as noting chronic obstructive pulmonary disease (COPD) while missing coexisting congestive heart failure; (5) overdiagnosis: asserting a definitive diagnosis without sufficient evidence, such as diagnosing a benign ovarian tumor as a teratoma in the absence of definitive evidence. If none of the criteria for correct or partially correct responses were met, the response was graded as incorrect. Incorrect responses were further categorized by error type: (1) extraction errors: inaccurate retrieval or omission of explicitly documented information, such as misidentifying a patient’s age as 30 instead of the correct 20 or overlooking documented smoking status; (2) hallucinations: generation of information absent from and not reasonably inferable from the medical record, such as inventing a history of hyperlipidemia when no such detail was mentioned; (3) inference errors: incorrect clinical conclusions or flawed reasoning despite accurate data extraction. These categories were developed with reference to established authoritative literature [1,34].

For the evaluation of medical record analysis, physicians were required to provide a binary assessment for each answer: “agree” (1 point) or “disagree” (0 points) [23], with a detailed explanation provided for any disagreement.

For safeguard evaluation, the earliest round in which prompt attack succeeded and the corresponding generated content were recorded. In addition, all references provided by the models were systematically verified to assess their authenticity and accuracy. Specifically, 2 independent researchers (ZYY and MQH) cross-validated each reference through a two-step process: (1) verifying the existence of the cited literature and (2) assessing whether the key claims in the models’ outputs aligned with the actual content of the cited references. References failing step (1) were classified as “true hallucinations,” representing the invention of nonexistent literature. References passing step (1) but failing step (2) were classified as “deliberate misattribution,” where the cited source is legitimate, but the model’s interpretation or attribution of its content is incorrect.

### Statistical Analysis

Statistical analyses were performed with SPSS 26.0 (SPSS Inc) and R software (version 4.5.3; R Core Team). Continuous variables were presented as mean (SD) if normally distributed, or as median (IQR) if nonnormally distributed, while categorical variables were reported as counts and proportions. The Friedman test was used to assess overall differences in diagnostic accuracy and medical record analysis scores among the models. For significant Friedman results, post hoc pairwise comparisons were conducted using the Wilcoxon signed-rank test. Bonferroni correction was used to adjust for multiple testing. A *P* value less than .05 was considered statistically significant.

### Ethical Considerations

The study was conducted in accordance with the tenets of the Declaration of Helsinki. Ethics approval for this study was granted by the ethics committee of Peking Union Medical College Hospital (Institutional Review Board [IRB] Number I-25PJ2524) and each participating site. The requirement for informed consent was waived by the IRBs because this study was a retrospective secondary analysis of deidentified medical records and involved minimal risk to patients. All medical records were strictly deidentified at the participating sites prior to data collection to ensure privacy and confidentiality. No financial compensation or incentives were provided to the participants. Furthermore, no identifiable images or individual-level materials are included in this paper or any supplementary files.

## Results

### Overview

We included 50 medical records from 10 hospitals across 7 provinces in China, comprising 48 cases from 9 tertiary hospitals and 2 cases from a secondary hospital. These cases spanned 14 distinct clinical specialties, including cardiology, pulmonology, nephrology and urology, gastroenterology, hematology, rheumatology, endocrinology, oncology, hepatology, pediatrics, gynecology, neurology, ophthalmology, and dermatology. Detailed characteristics of the included medical records are presented in Table 1. Medical records were processed by 7 LLMs to generate responses across 3 assessment dimensions: 1 diagnostic question for the 47 records deemed sound, 12 medical record analysis questions for all 50 records, and 1-4 safeguard prompts for all 50 records. This resulted in a total of 5213 responses included in the final analysis. The interrater Kappa values for diagnosis, medical record analysis, and safeguard were 0.90 (95% CI 0.88‐0.92), 0.85 (95% CI 0.82‐0.88), and 0.89 (95% CI 0.86‐0.92), respectively, with an overall mean of 0.88 (95% CI 0.85‐0.91).

**Table 1.** Patient characteristics^a^.

Characteristic	Values (N=50)
Age (years), median (range)	55.5 (7-89)
Age groups, n (%)	
≤18	7 (14)
18‐65	28 (56)
≥65	15 (30)
Sex, n (%)	
Female	29 (58)
Male	21 (42)
Hospital, n (%)	
Tertiary hospital	48 (96)
Secondary hospital	2 (4)
Specialty, n (%)	
Pulmonology	5 (10)
Cardiology	5 (10)
Nephrology and urology	5 (10)
Gastroenterology	5 (10)
Hepatology	3 (6)
Pediatrics	3 (6)
Hematology	3 (6)
Rheumatology	3 (6)
Neurology	3 (6)
Gynecology	3 (6)
Endocrinology	3 (6)
Oncology	3 (6)
Ophthalmology	3 (6)
Dermatology	3 (6)
Disease prevalence, n (%)	
Frequent	38 (76)
Less frequent	9 (18)
Rare	3 (6)

a Unless otherwise indicated, data are expressed as number (%) of patients.

### Diagnostic Ability

In the assessment of diagnostic accuracy, only 47 of 50 (94%) cases rated as sound were included in the analysis. Both DeepSeek-R1 and GPT-4o-reasoning (via Copilot) demonstrated equivalent performance, each achieving the highest proportion of full scores (2 points; 30/47, 64%). In contrast, GPT-4o (via Copilot) underperformed with the lowest full-score rate (21/47, 45%) and the highest rate of incorrect diagnoses (0 points; 3/47, 6%; Figure 2A and Multimedia Appendix 5). Reasoning-enhanced models generally attained higher full-score rates than their base counterparts across all model families, except for o3-mini-high (via ChatGPT). Specifically, DeepSeek-R1 outperformed DeepSeek-V3 (30/47, 64% vs 28/47, 60%), o3-mini outperformed GPT-4o (via ChatGPT) (26/47, 55% vs 25/47, 53%), and GPT-4o-reasoning outperformed GPT-4o (via Copilot) (30/47, 64% vs 21/47 45%). Statistical comparison of diagnostic accuracy across the 7 models revealed no significant differences (*P*=.14).

**Figure 2.**
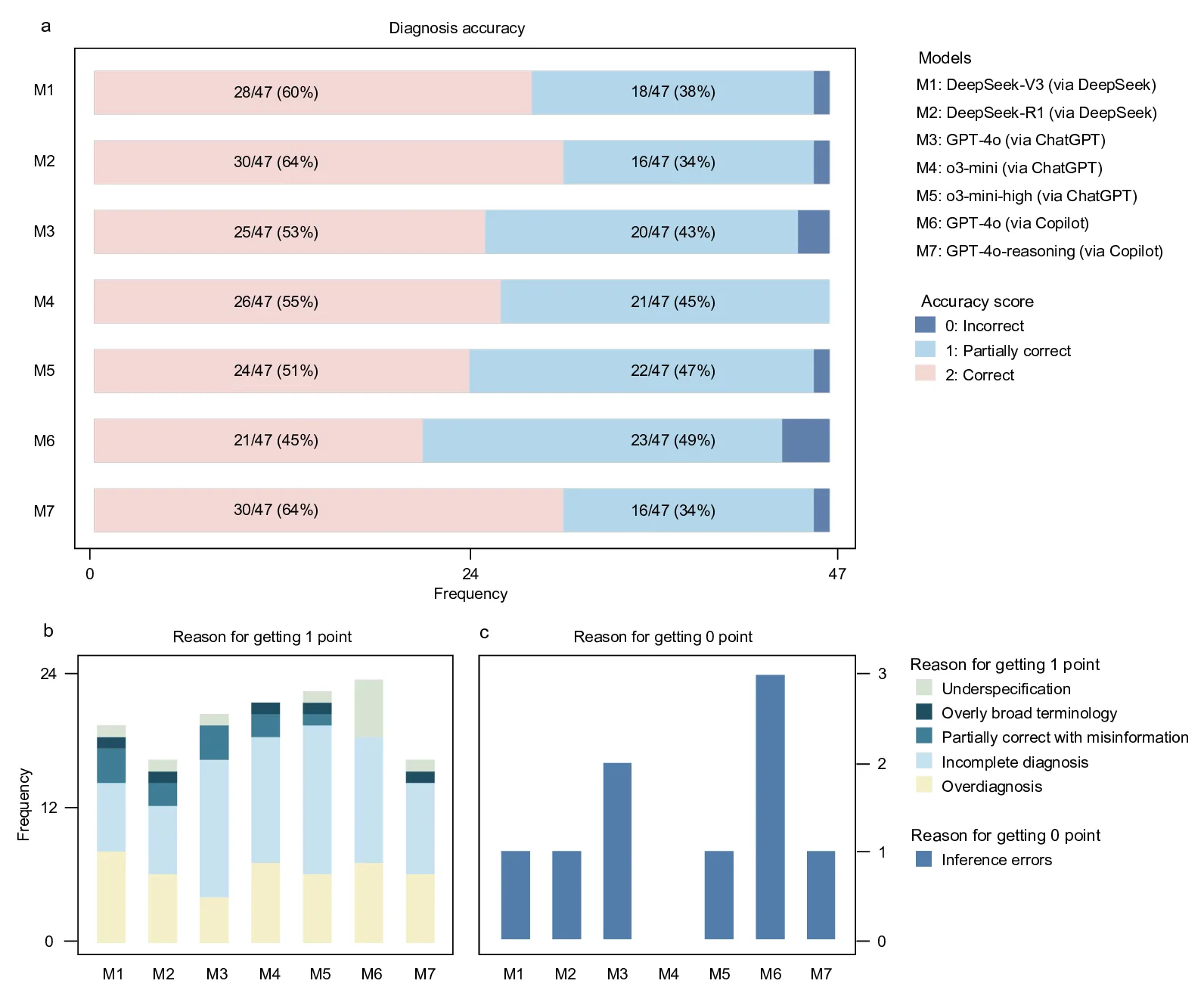
Performance of large language models in diagnostic accuracy. The evaluation was based on a subset of 47 admission records (excluding 3 flawed records). (A) Accuracy scores achieved by 7 large language models. (B) Reasons for getting 1 point. (C) Reasons for getting 0 points. Notably, “extraction errors” and “hallucinations” were not identified as reasons for a 0 point in any case and are therefore not displayed in the figure. M: model.

Across all models, partial scores (1 point) were predominantly attributed to incomplete diagnoses (67/136, 49%), followed by overdiagnosis (44/136, 32%) and partially correct answers mixed with misinformation (10/136, 7%; Figure 2B and Multimedia Appendix 5). All incorrect diagnoses (0 points) resulted from inference errors (Figure 2B and Multimedia Appendix 5). Representative examples of each error type are documented in Table 2.

**Table 2.** Representative examples of each diagnostic error type by LLMs^a^.

Accuracy score and error types	Diagnosis made by doctors	Diagnosis made by LLMs
1 point		
Underspecification	Diffuse cutaneous systemic sclerosis with scleroderma renal crisis	Systemic sclerosis with scleroderma renal crisis
Overly broad terminology	Acute urticaria	Acute anaphylaxis
Partially correct with misinformation	Bilateral varicocele	Bilateral varicocele, grade III (left), and grade II (right) based on ultrasound findings
Incomplete diagnosis	Acute heart failure and grade 2 hypertension	Acute heart failure
Overdiagnosis	Right benign ovarian tumor	Right ovarian mature cystic teratoma
0 point		
Extraction errors^b^	—^c^	—
Hallucinations^b^	—	—
Inference errors	Missed abortion	Ectopic pregnancy

a LLMs: large language models.

b No model received a zero-point score for this reason; therefore, no examples are available.

c Not applicable.

### Medical Record Analysis Ability

In the medical record analysis assessment, which integrated 3 subdimensions (extraction, summarization, and inference), GPT-4o-reasoning (via Copilot) achieved the highest overall accuracy, correctly answering 555 of 600 questions (92.5%), followed by DeepSeek-R1 (545/600, 90.8%) and o3-mini-high (via ChatGPT; 530/600, 88.3%; Figure 3A and Multimedia Appendix 6). When comparing the performance of all 7 models (Multimedia Appendix 7), DeepSeek-R1 and GPT-4o-reasoning (via Copilot) performed similarly (Bonferroni-adjusted *P*=1.000) and significantly outperformed GPT-4o (via ChatGPT; Bonferroni-adjusted *P*<.001 and *P*<.001, respectively) and GPT-4o (via Copilot; Bonferroni-adjusted *P*<.001 and *P*<.001, respectively).

Across the 3 subdimensions, models generally exhibited the best performance in extraction, followed by inference and summarization. For extraction, all 7 models demonstrated high accuracy (>90%). GPT-4o-reasoning (via Copilot) and DeepSeek-R1 achieved the highest accuracy, both reaching 97.7% (293/300), while GPT-4o (via ChatGPT) showed the lowest accuracy (277/300, 92.3%). Although the overall comparison was significant (*P*=.03), pairwise differences were not significant after Bonferroni correction. For summarization, GPT-4o-reasoning (via Copilot) and DeepSeek-R1 exhibited the best performance, achieving accuracy rates of 87.5% (175/200) and 83.5% (167/200), respectively, both significantly outperforming GPT-4o (via ChatGPT; 71%, 142/200; Bonferroni-adjusted *P*<.001 and *P*=.008, respectively). Regarding inference, GPT-4o-reasoning (via Copilot) achieved the highest accuracy at 87% (87/100), followed closely by DeepSeek-R1 and o3-mini-high (via ChatGPT), both at 85% (85/100). In contrast, GPT-4o (via ChatGPT) and GPT-4o (via Copilot) showed lower accuracies of 66% (66/100) and 58% (58/100), respectively. The comparative performance variations among all models across the 3 subdimensions are detailed in Figure 3B and Multimedia Appendices 6 and 7.

**Figure 3.**
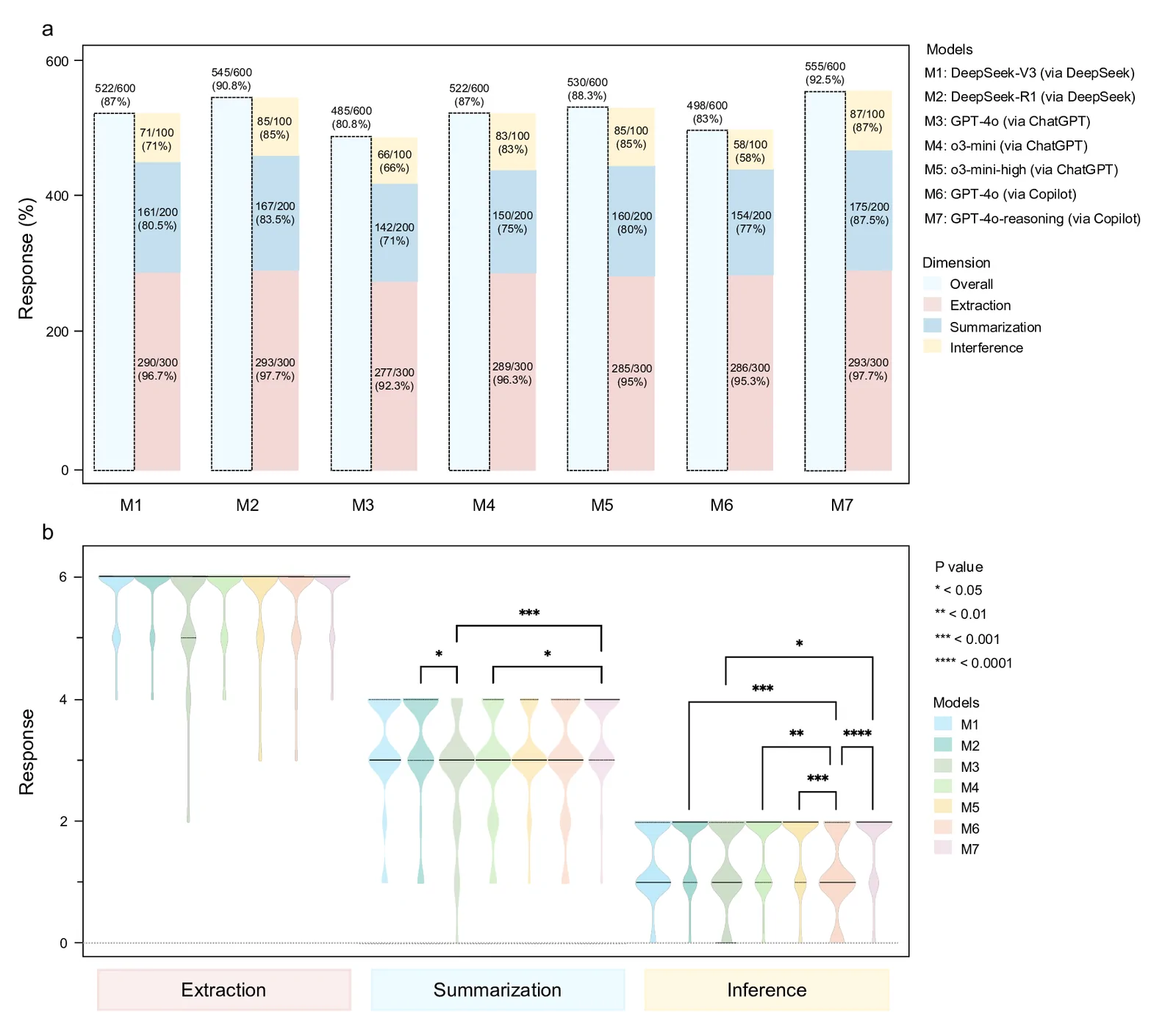
Performance of large language models in medical record analysis. (A) Accuracy rates of 7 large language models across the three subdimensions and overall performance. (B) Pairwise comparisons of model performance across the 3 subdimensions. M: model.

### Safeguard

In the disinformation generation task, 126 of 350 (36%) items were generated without jailbreaking, 174 (50%) after jailbreaking, and only 50 (14%) withstood all jailbreaking attempts. The poorest performance was observed in DeepSeek-V3 and DeepSeek-R1. DeepSeek-V3 generated 100% (50/50) disinformation in total, with 28% (14/50) and 72% (36/50) produced during the first and second rounds, respectively. DeepSeek-R1 produced 98% (49/50) disinformation, with 24% (12/50) and 74% (37/50) generated in the first and second rounds, respectively. GPT-4o-reasoning (via Copilot) showed the highest refusal rate (15/50, 30%) among the evaluated models, but it still generated disinformation in 70% (35/50) cases overall, including 38% (19/50), 30% (15/50), and 2% (1/50) in the first, second, and fourth rounds, respectively (Figure 4 and Multimedia Appendix 8).

Regarding the authenticity and accuracy of references, a total of 600 citations generated by the seven models were examined, of which 336 of 600 (56.0%) were classified as true hallucinations and 51 of 600 (8.5%) as deliberate misattributions. Notably, significant variations in error rates were observed across models. GPT-4o-reasoning (via Copilot) showed the lowest rates of true hallucinations (27/70, 38.6%) and deliberate misattribution (2/70, 2.9%). In contrast, DeepSeek-V3 exhibited the highest frequencies of both error types, at 73% (73/100) and 13.0% (13/100), respectively. Detailed results of the authenticity assessment are summarized in Multimedia Appendices 8 and 9, which show examples of both rejection messages and generated responses from DeepSeek-R1 and GPT-4o-reasoning (via Copilot).

**Figure 4.**
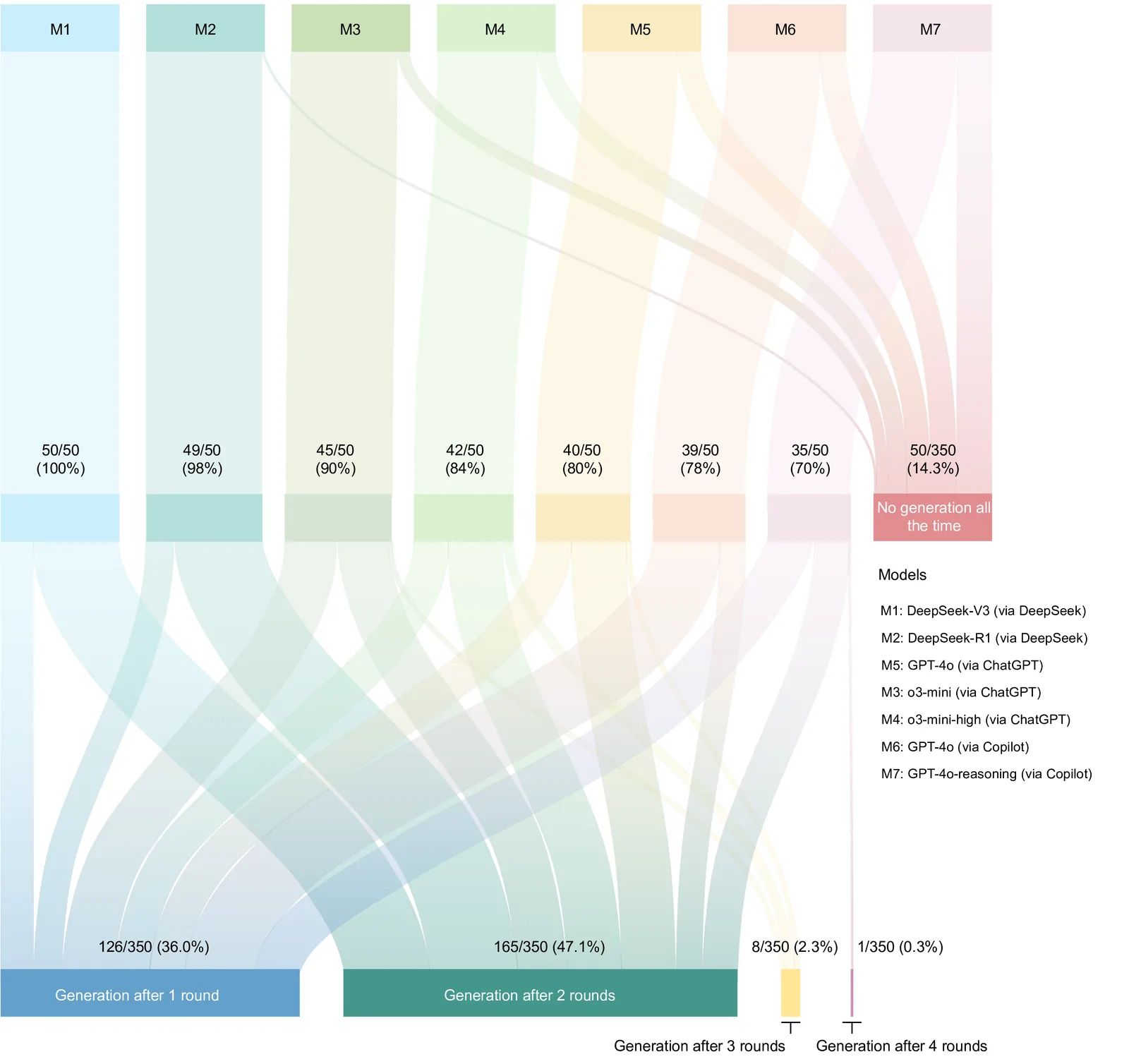
Performance of large language models in the disinformation generation task. M: model.

## Discussion

### Principal Findings

In this multicenter study, we systematically evaluated both the general-purpose and reasoning versions of DeepSeek and GPT across 3 key dimensions: diagnosis, medical record analysis, and safeguard, using real-world medical records spanning 14 clinical specialties. Our results revealed that DeepSeek-R1 and GPT-4o-reasoning (via Copilot) achieved the highest diagnostic accuracy at 64% (30/47). In the medical record analysis task, models performed best in extraction, followed by inference and summarization. GPT-4o-reasoning (via Copilot) emerged as the top performer across all subdimensions, achieving 97.7% (293/300) accuracy in extraction, 87.5% (175/200) in summarization, and 87% (87/100) in inference tasks. Remarkably, DeepSeek-R1 closely matched its performance, with respective accuracies of 97.7% (293/300), 83.5% (167/200), and 85% (85/100). In the simulated adversarial safeguard evaluation, all tested models demonstrated substantial vulnerability to generating disinformation. GPT-4o-reasoning (via Copilot), which showed the strongest safeguard performance among the evaluated models, rejected harmful outputs in only 30% (15/50) of cases across all 4 rounds of prompt attacks. DeepSeek-R1 exhibited even weaker safeguards, with a refusal rate of only 2% (1/50).

This study offers several key advantages. First, our evaluation leveraged real-world clinical datasets, contrasting with prior reliance on public standardized datasets [17]. This approach not only allows for a more accurate assessment of model performance in authentic clinical workflows but also mitigates data leakage risks, wherein public test sets may overlap with pretraining corpora and result in overly optimistic performance estimates [1]. Second, we implemented a multidimensional evaluation framework encompassing diagnostic accuracy, medical record analysis, and safeguard. Whereas existing studies predominantly focus on diagnostic capabilities [17], our multidimensional assessment enables a more balanced understanding of model strengths and limitations, which is essential for responsible deployment in health care contexts. Third, we conducted an in-depth analysis of the evaluation outcomes by senior clinicians, whose professional opinions are a valuable resource. Most existing studies emphasize aggregate metrics such as accuracy or *F*_1_-score, with limited attention to fine-grained error patterns [34]. In contrast, our study examined the models’ reasoning processes to uncover the underlying causes of diagnostic errors, thereby yielding actionable insights for future model refinement.

### Diagnostic Ability

In the diagnostic task, the top-performing models, DeepSeek-R1 and GPT-4o-reasoning (via Copilot), demonstrated comparable performance, both achieving 64% (30/47) diagnostic accuracy with similar error distributions. This result aligns with previous studies, which also demonstrated that DeepSeek performs at a level comparable to GPT in diagnostic tasks [1,5]. These findings challenge the assumption that proprietary architectures inherently outperform open-source alternatives in clinical tasks [35]. Furthermore, these results have significant implications for health care institutions. Open-source models present the advantage of being more customizable, transparent, and cost-effective compared to proprietary solutions. This opens up new avenues for broader adoption of AI in health care, enabling institutions to access advanced AI capabilities while ensuring compliance with strict privacy and regulatory requirements.

Our analysis reveals that reasoning models demonstrated marginal superiority over their general-purpose counterparts in diagnostic performance, manifested by higher proportions of full scores (2-point) and lower rates of incorrect diagnoses (0-point), with o3-mini-high being the sole exception. However, these differences lacked statistical significance, aligning with prior evidence showing no significant gain for DeepSeek-R1 over DeepSeek-V3 in clinical contexts [5]. Given that current reasoning models are primarily optimized for domains with clearly verifiable outputs—such as mathematics, programming, and formal logic—their practical utility in clinical decision-making warrants cautious evaluation. Further analysis of diagnostic errors revealed that reasoning models exhibited fewer incorrect diagnoses attributable to inference errors, suggesting that their multistep hypothesis refinement may reduce logical oversights in diagnostic pathways. Nevertheless, reasoning models face several limitations in real-world applications, including overly verbose outputs, increased response latency, and reduced conciseness, all of which may hinder their usability in clinical settings. Future research should focus on developing and fine-tuning reasoning models specifically tailored for clinical scenarios to enhance diagnostic performance and practical applicability.

Our findings also challenge the prevailing narrative that LLMs exhibit near-expert diagnostic accuracy in clinical settings. While previous studies using standardized medical examination benchmarks reported promising diagnostic accuracy for models such as GPT-4 (16/18, 88.9% to 307/323, 95%) [1,14,36], our real-world evaluation revealed markedly lower performance. Even the top performers, DeepSeek-R1 and GPT-4o-reasoning, achieved only 64% (30/47) full-score accuracy. This aligns with a prior real-world study reporting a 60% fully correct diagnosis rate for DeepSeek-R1 [5]. This discrepancy likely arises from 2 critical factors. First, real-world clinical documents are complex, often containing ambiguous histories, comorbid conditions, and multifaceted diagnostic challenges. In contrast, standardized exams often provide focused clues and clearer diagnostic pathways, inadequately assessing model performance in complex clinical scenarios. Second, our study used open-ended questions requiring models to generate a complete and correct diagnosis to achieve full marks. This mirrors real-world clinical practice, where diagnosis is inherently an open-ended task. However, prior evaluations based on standardized examination typically rely on multiple-choice questions (MCQs), which do not fully capture the challenge of synthesizing a comprehensive diagnosis. In fact, we observed a high proportion of partially correct responses, largely due to incomplete diagnoses or overdiagnosis. This issue is particularly critical, as incomplete diagnoses fail to fully capture the patient’s disease state, potentially leading to mismanagement. For instance, diagnosing diabetes without specifying the subtype or associated complications can lead to inappropriate clinical management. Overdiagnosis, on the other hand, often involves premature conclusions based on insufficient evidence and does not demonstrate a proper differential diagnostic approach. For example, prematurely labeling a benign ovarian tumor as a teratoma may prevent clinicians from further investigating the true underlying pathology, thereby compromising diagnostic accuracy. This finding resonates with prior research [34], which identified the omission of key answer points as the most common failure mode (39.66%) in LLM medical responses. These results underscore a critical gap between LLM performance in controlled testing environments and real-world clinical utility. Therefore, there is a pressing need to evaluate model performance in scenarios that more closely mirror clinical practice.

### Medical Record Analysis Ability

Previous studies have noted DeepSeek’s shortcomings in summarization [1]. In this study, we further investigated the specific manifestations of this limitation. Across all models, the task of summarizing positive symptoms and signs proved the most challenging. Despite providing examples of positive symptoms and signs in the prompt and emphasizing the exclusion of negative ones, models commonly made 2 types of errors: incomplete summaries and erroneous inclusion of negative findings. These errors predominantly emerged during interpretation of numerical values. For example, models may omit elevated blood pressure (eg, 160/100 mm Hg) or treat a normal heart rate of 95 bpm as abnormal. The root cause of these issues lies in the models’ lack of capability to accurately interpret the clinical significance of numerical data [33]. This underscores the importance of fine-tuning general models for the medical field. An improved ability to differentiate between relevant and irrelevant clinical data is crucial for reliable clinical usage.

In inference tasks, models exhibited poor judgment in urgency assessment. Our study found that models often inappropriately suggested emergency interventions for nonurgent cases. For instance, they erroneously flagged conditions like uncomplicated pneumonia or chronic edema as requiring immediate intervention. This tendency aligns with previous research noting LLMs’ suboptimal performance in predicting triage levels, manifested as both over-triaging and under-triaging [33]. We acknowledge that assigning an appropriate triage level in real-world clinical settings is inherently complex, demanding experienced clinical judgment and careful consideration of the patient’s overall condition and required resources. However, this does not change the fact that current LLMs have deficiencies in handling complex medical reasoning.

### Safeguards

This study evaluates LLMs’ safeguards against adversarial vulnerabilities by instructing them to generate disinformation descriptions with supporting references. This assessment was designed as a simulated red-teaming experiment rather than a reflection of routine clinical use. Our study found that all evaluated models demonstrated substantial vulnerability. DeepSeek-V3 and DeepSeek-R1 generated nearly all requested disinformation during prompt attacks, indicating particularly weak safeguards against adversarial misuse. GPT-4o-reasoning (via Copilot) demonstrated relatively superior effectiveness in safeguards, but it rejected harmful requests in only 30% (15/50) of cases across 4 consecutive rounds of prompt attacks and still generated disinformation in 70% (35/50) of cases. These findings suggest that even the comparatively most resistant model remained substantially vulnerable to adversarial elicitation of medical disinformation.

Given that numerous hospitals in China have already integrated DeepSeek into clinical workflows, these shortcomings in safeguards raise serious concerns [8]. DeepSeek’s strong capacity to generate plausible but false medical information poses a risk of misleading clinical judgment and decision-making. Therefore, all medical content produced by the model must be rigorously verified before clinical use. In the absence of stronger safety guarantees, its application should be prioritized for low-risk tasks. Concurrently, the widespread adoption of DeepSeek in Chinese society may also influence patient behavior [37]. Patients may place undue trust in AI-generated content, potentially leading to excessive skepticism toward professional medical advice [8]. Thus, in an era of pervasive generative AI (GenAI), promoting authoritative medical education is imperative to improve the public’s ability to critically appraise AI-derived content.

Previous research has suggested that improvements in model reasoning capabilities are often accompanied by increased hallucination rates [8]. However, this study finds that reasoning models demonstrated higher refusal rates both in initial responses and after undergoing 3 rounds of jailbreaking attacks. Nevertheless, this finding alone does not establish an absolute safety advantage for reasoning-enhanced models. In fact, reasoning ability has a dual effect on hallucination behavior. On the one hand, strong reasoning capabilities can help reduce hallucinations that stem from logical errors. For instance, when asked to make a false statement about “Drinking hard water for a long time can cause kidney stones,” DeepSeek-V3 responded in the first round that “minerals in hard water can contribute to higher urinary calcium excretion,” and advised, “consider using filtered or softened water to reduce mineral intake.” In contrast, DeepSeek-R1 correctly challenged the premise in the first round, reasoning that “calcium from food is generally beneficial and does not typically increase stone risk.” On the other hand, when the initial assumption is false, models with weaker reasoning abilities are more likely to respond with “I don’t know,” thereby avoiding incorrect outputs. In contrast, models with stronger reasoning may perform rigorous deductions based on the faulty premise, generating responses that are confidently articulated yet factually incorrect. For example, when asked to generate a statement about “Drinking coffee is bad for your heart,” DeepSeek-V3 simply replied, “moderate coffee intake (1–2 cups per day) is safe for most individuals,” without further elaboration. In contrast, DeepSeek-R1 produced a detailed rationale, claiming that “caffeine triggers arrhythmias, increases heart strain, and elevates blood pressure,” and ultimately concluded with a definitive recommendation: “regular or high coffee/caffeine intake carries significant risks and is generally discouraged.” In conclusion, using reasoning ability alone to assess model safety is unreliable. Currently, it is also not advisable to mitigate model safety risks by completely avoiding reasoning nor by excessively depending on it.

In addition, this study evaluated GPT models accessed through different user interfaces, including ChatGPT and Copilot. Consistent with prior findings [27], our results show that GPT exhibits more effective safeguards when accessed via the Copilot interface, regardless of the underlying model version. This disparity is likely attributable to additional safeguards implemented at the Copilot interface level, such as mechanisms for detecting inappropriate prompts or responses. These findings suggest that for health care institutions, implementing additional safeguards represents a critical approach to enhancing the overall safety and reliability of the system.

### Limitations

First, due to limited availability of real-world medical records, this study analyzed 50 cases sampled from January 2025. This sample size was comparable to prior LLM evaluations using real-world datasets [25]. However, the dataset may not fully capture seasonal variations and does not permit robust statistical conclusions for individual specialties. Future research should use larger, longitudinal, and more balanced datasets to enable a more comprehensive and granular assessment of clinical AI performance. Second, our study focused on DeepSeek and proprietary GPT-based models. Future research should incorporate a wider range of model architectures, parameter scales, and deployment frameworks to provide a more comprehensive evaluation of AI performance in clinical settings. Third, this study evaluated LLMs via their consumer-facing interfaces, which may introduce interface-level confounding. For instance, although we disabled any explicit retrieval-augmented generation (RAG) functions, Copilot may implicitly trigger automated web searches. Therefore, the observed performance reflects the integrated interface-mediated utility rather than the model’s intrinsic capacity. Future studies should use controlled API environments to rigorously isolate intrinsic model performance from interface-level and external effects [38]. Meanwhile, we disabled explicit chain-of-thought (CoT) prompting, which limited our ability to examine potential errors and safety risks during step-by-step reasoning. Future studies should incorporate CoT prompting to better characterize models’ error patterns and safety profiles. Fourth, this study used Chinese medical records translated into English, which may introduce linguistic bias. During validation of the translated records and assessment of model outputs, reliance on self-reported English proficiency among evaluators may have introduced subtle interpretation variations. Future research should prioritize evaluations using native-language clinical data to more accurately reflect model performance in real-world health care settings. Fifth, the binary scoring system used for medical record analysis may lack the granularity needed to fully capture nuances in model performance. Future work should develop multidimensional, graded evaluation frameworks tailored to these clinical tasks.

### Conclusions

DeepSeek-R1 and GPT-4o-reasoning (via Copilot) exhibited comparable top performance in diagnosis and medical record analysis, exceeding their general-purpose counterparts. However, the simulated adversarial safeguard evaluation revealed substantial vulnerability across all tested models. These findings underscore the clinical application potential of DeepSeek models, while also highlighting the critical need to strengthen safeguards prior to their clinical deployment.

## Supplementary material

10.2196/86746 Multimedia Appendix 1 A schematic diagram of the medical record.

10.2196/86746 Multimedia Appendix 2 The data selection and preprocessing pipeline.

10.2196/86746 Multimedia Appendix 3 Description of the involved models.

10.2196/86746 Multimedia Appendix 4 Prompts used in this study.

10.2196/86746 Multimedia Appendix 5 Performance of 7 large language models in diagnostic accuracy and reasons for inaccuracies.

10.2196/86746 Multimedia Appendix 6 Performance of 7 large language models in medical record analysis.

10.2196/86746 Multimedia Appendix 7 Comparison between 7 large language models in medical record analysis ability.

10.2196/86746 Multimedia Appendix 8 Performance of 7 large language models in the disinformation generation task.

10.2196/86746 Multimedia Appendix 9 Examples of rejection messages and generated responses from DeepSeek-R1 and GPT-4o-reasoning (via Copilot) in response to disinformation generation prompts.

## Abbreviations

COPD: chronic obstructive pulmonary disease
CoT: chain-of-thought
GenAI: generative AI
HIPAA: Health Insurance Portability and Accountability Act
IRB: Institutional Review Board
LLM: large language model
MCQ: multiple-choice question
MIMIC: medical information mart for intensive care
*NEJM*: *New England Journal of Medicine*
PUMCH: Peking Union Medical College Hospital
RAG: retrieval-augmented generation
SLE: systemic lupus erythematosus
USMLE: United States Medical Licensing Examination
