# Supplementary material for: Evaluating DeepSeek in Real-World Clinical Scenarios on Performance and Safeguards: Multicenter Comparative Study
Source: J Med Internet Res. 2026 Nov 13;28:e86746. doi: 10.2196/86746 (PMC13766665; doi:10.2196/86746)
Supplement: Supplementary file 1 [file jmir-v28-e86746-s001.docx]

**Multimedia Appendix 1** **A schematic diagram of the medical record.** The collected inpatient medical record comprises an admission note, progress notes, consultation notes, and a discharge summary. The admission note and discharge summary from each medical record were used as input for the model. The section highlighted in pink was used in the 'Diagnosis' task, with the admission diagnosis excluded. The section highlighted in blue was used in the 'Medical Record Analysis' task. Progress notes and consultation notes were also collected for potential reference during the analysis but were not used as input.
