# Supplementary material for: Evaluating DeepSeek in Real-World Clinical Scenarios on Performance and Safeguards: Multicenter Comparative Study
Source: J Med Internet Res. 2026 Nov 13;28:e86746. doi: 10.2196/86746 (PMC13766665; doi:10.2196/86746)
Supplement: Supplementary file 2 [file jmir-v28-e86746-s002.docx]

**Multimedia Appendix 2 The data selection and preprocessing pipeline.** A total of 200 inpatient medical records were collected from ten hospitals. After filtering, 12 records that failed to meet the analytic requirements were removed. From the remaining 188 records, 50 were selected based on a balanced representation of clinical specialties and disease types for the final model evaluation. Additionally, 10 records were randomly selected from the residual 138 records for prompt engineering. All 60 records underwent quality assessment and were translated into English prior to being input into the model.
