# Supplementary material for: Evaluating DeepSeek in Real-World Clinical Scenarios on Performance and Safeguards: Multicenter Comparative Study
Source: J Med Internet Res. 2026 Nov 13;28:e86746. doi: 10.2196/86746 (PMC13766665; doi:10.2196/86746)
Supplement: Supplementary file 3 [file jmir-v28-e86746-s003.docx]

**Multimedia Appendix 3 Description of the involved models.**

| **Models** | **Company** | **Advantages** | **Enhanced reasoning** | **Open Source** | **Website** |
| --- | --- | --- | --- | --- | --- |
| **DeepSeek-V3**  (via DeepSeek) | DeepSeek | Optimized for multilingual environments; Featuring robust long-context processing capabilities. | × | √ | [https://www.deepseek.com](https://www.deepseek.com/) |
| **DeepSeek-R1**  (via DeepSeek) | DeepSeek | Open-source and customizable to ensure compliance with medical privacy regulations; Enhanced reasoning abilities to streamline clinical decision-making logic; High cost-effectiveness to facilitate the widespread adoption of medical AI. | √ | √ | [https://www.deepseek.com](https://www.deepseek.com/) |
| **GPT-4o**  (via ChatGPT) | OpenAI | Supports arbitrary combinations of text, audio, and image as inputs and outputs, with rapid response times. | × | × | [https://chat.openai.com](https://chat.openai.com/) |
| **o3-mini**  (via ChatGPT) | OpenAI | A lightweight, efficient version. Offering enhanced reasoning capabilities, fast reasoning speed, and low resource consumption. | √ | × | [https://chat.openai.com](https://chat.openai.com/) |
| **o3-mini-high**  (via ChatGPT) | OpenAI | The o3-mini high-performance derivative, which builds upon the lightweight foundation to further elevate reasoning capabilities. | √ | × | [https://chat.openai.com](https://chat.openai.com/) |
| **GPT-4o**  (via Copilot) | Microsoft | Accessible via the Copilot interface, with strong default safety filters and conservative outputs, suited for sensitive medical scenarios. | × | × | [https://copilot.microsoft.com](https://copilot.microsoft.com/) |
| **GPT-4o-reasoning**  (via Copilot) | Microsoft | Enhanced reasoning mode that markedly improves logical analysis capabilities, specifically optimized for tackling complex problems. | √ | × | [https://copilot.microsoft.com](https://copilot.microsoft.com/) |
