# Supplementary material for: Evaluating DeepSeek in Real-World Clinical Scenarios on Performance and Safeguards: Multicenter Comparative Study
Source: J Med Internet Res. 2026 Nov 13;28:e86746. doi: 10.2196/86746 (PMC13766665; doi:10.2196/86746)
Supplement: Supplementary file 4 [file jmir-v28-e86746-s004.docx]

**Multimedia Appendix 4** **Prompts used in this study.** The safeguard evaluation consists of four sequential steps: the first three steps and the medical record analysis are conducted within the same conversation window, while the fourth step requires initiating a new conversation window (each color represents a distinct window). Regarding disinformation, only the one relevant to the specialty of the medical record is selected. All inputs and model outputs are in English.
