# Supplementary material for: Evaluating DeepSeek in Real-World Clinical Scenarios on Performance and Safeguards: Multicenter Comparative Study
Source: J Med Internet Res. 2026 Nov 13;28:e86746. doi: 10.2196/86746 (PMC13766665; doi:10.2196/86746)
Supplement: Supplementary file 5 [file jmir-v28-e86746-s005.docx]

**Multimedia Appendix 5 Performance of seven large language models in diagnostic accuracy and reasons for inaccuracies.**

|  | **Overall** | **DeepSeek-V3**  (via DeepSeek) | **DeepSeek-R1**  (via DeepSeek) | **GPT-4o**  (via ChatGPT) | **o3-mini**  (via ChatGPT) | **o3-mini-high**  (via ChatGPT) | **GPT-4o**  (via Copilot) | **GPT-4o-reasoning**  (via Copilot) |
| --- | --- | --- | --- | --- | --- | --- | --- | --- |
| ***Diagnosis accuracy score*** | | | | | | | | |
| **2, n (%)** | 184 (55.9) | 28 (59.6) | 30 (63.8) | 25 (53.2) | 26 (55.3) | 24 (51.1) | 21 (44.7) | 30 (63.8) |
| **1, n (%)** | 136 (41.3) | 18 (38.3) | 16 (34.0) | 20 (42.6) | 21 (44.7) | 22 (46.8) | 23 (48.9) | 16 (34.0) |
| **0, n (%)** | 9 (2.7) | 1 (2.1) | 1 (2.1) | 2 (4.3) | 0 | 1 (2.1) | 3 (6.4) | 1 (2.1) |
| ***Reasons for getting 1 point*** | | | | | | | | |
| **Underspecification, n (%)** | 10 (7.4) | 1 (5.6) | 1 (6.3) | 1 (5.0) | 0 | 1 (4.5) | 5(21.7) | 1 (6.3) |
| **Overly broad terminology, n (%)** | 5 (3.7) | 1 (5.6) | 1 (6.3) | 0 | 1 (4.8) | 1 (4.5) | 0 | 1 (6.3) |
| **Partially correct with misinformation, n (%)** | 10 (7.4) | 2 (11.1) | 2 (12.5) | 3 (15.0) | 2 (9.5) | 1 (4.5) | 0 | 0 |
| **Incomplete diagnosis, n (%)** | 67 (49.3) | 6 (33.3) | 6 (37.5) | 12 (60.0) | 11 (52.4) | 13 (59.1) | 11(47.8) | 8 (50.0) |
| **Overdiagnosis, n (%)** | 44 (32.4) | 8 (44.4) | 6 (37.5) | 4 (20.0) | 7 (33.3) | 6 (27.3) | 7(30.4) | 6 (37.5) |
| ***Reasons for getting 0 point*** | | | | | | | | |
| **Extraction errors, n (%)** | 0 | 0 | 0 | 0 | 0 | 0 | 0 | 0 |
| **Hallucinations, n (%)** | 0 | 0 | 0 | 0 | 0 | 0 | 0 | 0 |
| **Inference errors, n (%)** | 9(100.0) | 1(100.0) | 1(100.0) | 2(100.0) | 0 | 1(100.0) | 3(100.0) | 1(100.0) |
