# Supplementary material for: Evaluating DeepSeek in Real-World Clinical Scenarios on Performance and Safeguards: Multicenter Comparative Study
Source: J Med Internet Res. 2026 Nov 13;28:e86746. doi: 10.2196/86746 (PMC13766665; doi:10.2196/86746)
Supplement: Supplementary file 6 [file jmir-v28-e86746-s006.docx]

**Multimedia Appendix 6 Performance of seven large language models in medical record analysis.**

|  | **Overall, median (IQR)** | **Extraction, median (IQR)** | **Summarization, median (IQR)** | **Inference, median (IQR)** |
| --- | --- | --- | --- | --- |
| **DeepSeek-V3 (via DeepSeek)** | 11 (10-11) | 6 (6-6) | 3 (3-4) | 1 (1-2) |
| **DeepSeek-R1 (via DeepSeek)** | 11 (10-12) | 6 (6-6) | 4 (3-4) | 2 (1-2) |
| **GPT-4o (via ChatGPT)** | 10 (9-11) | 6 (5-6) | 3 (3-3) | 1 (1-2) |
| **o3-mini (via ChatGPT)** | 11 (10-11) | 6 (6-6) | 3 (3-4) | 2 (1-2) |
| **o3-mini-high (via ChatGPT)** | 11 (10-11) | 6 (5-6) | 3 (3-4) | 2 (1-2) |
| **GPT-4o (via Copilot)** | 10 (9-11) | 6 (6-6) | 3 (3-4) | 1 (1-2) |
| **GPT-4o-reasoning (via Copilot)** | 11 (11-12) | 6 (6-6) | 4 (3-4) | 2 (2-2) |

**Abbreviations:** IQR, interquartile range.
