# Supplementary material for: Evaluating DeepSeek in Real-World Clinical Scenarios on Performance and Safeguards: Multicenter Comparative Study
Source: J Med Internet Res. 2026 Nov 13;28:e86746. doi: 10.2196/86746 (PMC13766665; doi:10.2196/86746)
Supplement: Supplementary file 7 [file jmir-v28-e86746-s007.docx]

**Multimedia Appendix 7 Comparison between seven large language models in medical record analysis ability.**

| **Dimension** | **Model 1** | **Model 2** | ***P*-value** | **Bonferroni-adjusted *P*-value** |
| --- | --- | --- | --- | --- |
| **Overall** | DeepSeek-V3 (via DeepSeek) | DeepSeek-R1 (via DeepSeek) | 0.004 | 0.084 |
|  | DeepSeek-V3 (via DeepSeek) | GPT-4o (via ChatGPT) | **<0.001** | **0.017** |
|  | DeepSeek-V3 (via DeepSeek) | o3-mini (via ChatGPT) | 0.976 | 1.000 |
|  | DeepSeek-V3 (via DeepSeek) | o3-mini-high (via ChatGPT) | 0.338 | 1.000 |
|  | DeepSeek-V3 (via DeepSeek) | GPT-4o (via Copilot) | 0.014 | 0.294 |
|  | DeepSeek-V3 (via DeepSeek) | GPT-4o-reasoning (via Copilot) | **<0.001** | **0.010** |
|  | DeepSeek-R1 (via DeepSeek) | GPT-4o (via ChatGPT) | **<0.001** | **<0.001** |
|  | DeepSeek-R1 (via DeepSeek) | o3-mini (via ChatGPT) | 0.029 | 0.609 |
|  | DeepSeek-R1 (via DeepSeek) | o3-mini-high (via ChatGPT) | 0.232 | 1.000 |
|  | DeepSeek-R1 (via DeepSeek) | GPT-4o (via Copilot) | **<0.001** | **<0.001** |
|  | DeepSeek-R1 (via DeepSeek) | GPT-4o-reasoning (via Copilot) | 0.476 | 1.000 |
|  | GPT-4o (via ChatGPT) | o3-mini (via ChatGPT) | **<0.001** | **0.006** |
|  | GPT-4o (via ChatGPT) | o3-mini-high (via ChatGPT) | **<0.001** | **0.003** |
|  | GPT-4o (via ChatGPT) | GPT-4o (via Copilot) | 0.366 | 1.000 |
|  | GPT-4o (via ChatGPT) | GPT-4o-reasoning (via Copilot) | **<0.001** | **<0.001** |
|  | o3-mini (via ChatGPT) | o3-mini-high (via ChatGPT) | 0.552 | 1.000 |
|  | o3-mini (via ChatGPT) | GPT-4o (via Copilot) | 0.017 | 0.357 |
|  | o3-mini (via ChatGPT) | GPT-4o-reasoning (via Copilot) | **<0.001** | **0.018** |
|  | o3-mini-high (via ChatGPT) | GPT-4o (via Copilot) | 0.003 | 0.063 |
|  | o3-mini-high (via ChatGPT) | GPT-4o-reasoning (via Copilot) | 0.011 | 0.231 |
|  | GPT-4o (via Copilot) | GPT-4o-reasoning (via Copilot) | **<0.001** | **<0.001** |
| **Extraction** | DeepSeek-V3 (via DeepSeek) | DeepSeek-R1 (via DeepSeek) | 0.351 | 1.000 |
|  | DeepSeek-V3 (via DeepSeek) | GPT-4o (via ChatGPT) | 0.019 | 0.399 |
|  | DeepSeek-V3 (via DeepSeek) | o3-mini (via ChatGPT) | 0.830 | 1.000 |
|  | DeepSeek-V3 (via DeepSeek) | o3-mini-high (via ChatGPT) | 0.499 | 1.000 |
|  | DeepSeek-V3 (via DeepSeek) | GPT-4o (via Copilot) | 0.336 | 1.000 |
|  | DeepSeek-V3 (via DeepSeek) | GPT-4o-reasoning (via Copilot) | 0.437 | 1.000 |
|  | DeepSeek-R1 (via DeepSeek) | GPT-4o (via ChatGPT) | 0.003 | 0.063 |
|  | DeepSeek-R1 (via DeepSeek) | o3-mini (via ChatGPT) | 0.336 | 1.000 |
|  | DeepSeek-R1 (via DeepSeek) | o3-mini-high (via ChatGPT) | 0.098 | 1.000 |
|  | DeepSeek-R1 (via DeepSeek) | GPT-4o (via Copilot) | 0.096 | 1.000 |
|  | DeepSeek-R1 (via DeepSeek) | GPT-4o-reasoning (via Copilot) | 1.000 | 1.000 |
|  | GPT-4o (via ChatGPT) | o3-mini (via ChatGPT) | 0.021 | 0.441 |
|  | GPT-4o (via ChatGPT) | o3-mini-high (via ChatGPT) | 0.144 | 1.000 |
|  | GPT-4o (via ChatGPT) | GPT-4o (via Copilot) | 0.133 | 1.000 |
|  | GPT-4o (via ChatGPT) | GPT-4o-reasoning (via Copilot) | 0.006 | 0.126 |
|  | o3-mini (via ChatGPT) | o3-mini-high (via ChatGPT) | 0.374 | 1.000 |
|  | o3-mini (via ChatGPT) | GPT-4o (via Copilot) | 0.428 | 1.000 |
|  | o3-mini (via ChatGPT) | GPT-4o-reasoning (via Copilot) | 0.408 | 1.000 |
|  | o3-mini-high (via ChatGPT) | GPT-4o (via Copilot) | 0.842 | 1.000 |
|  | o3-mini-high (via ChatGPT) | GPT-4o-reasoning (via Copilot) | 0.159 | 1.000 |
|  | GPT-4o (via Copilot) | GPT-4o-reasoning (via Copilot) | 0.120 | 1.000 |
| **Summarization** | DeepSeek-V3 (via DeepSeek) | DeepSeek-R1 (via DeepSeek) | 0.368 | 1.000 |
|  | DeepSeek-V3 (via DeepSeek) | GPT-4o (via ChatGPT) | 0.004 | 0.084 |
|  | DeepSeek-V3 (via DeepSeek) | o3-mini (via ChatGPT) | 0.117 | 1.000 |
|  | DeepSeek-V3 (via DeepSeek) | o3-mini-high (via ChatGPT) | 0.856 | 1.000 |
|  | DeepSeek-V3 (via DeepSeek) | GPT-4o (via Copilot) | 0.373 | 1.000 |
|  | DeepSeek-V3 (via DeepSeek) | GPT-4o-reasoning (via Copilot) | 0.047 | 0.987 |
|  | DeepSeek-R1 (via DeepSeek) | GPT-4o (via ChatGPT) | **<0.001** | **0.008** |
|  | DeepSeek-R1 (via DeepSeek) | o3-mini (via ChatGPT) | 0.029 | 0.609 |
|  | DeepSeek-R1 (via DeepSeek) | o3-mini-high (via ChatGPT) | 0.298 | 1.000 |
|  | DeepSeek-R1 (via DeepSeek) | GPT-4o (via Copilot) | 0.090 | 1.000 |
|  | DeepSeek-R1 (via DeepSeek) | GPT-4o-reasoning (via Copilot) | 0.362 | 1.000 |
|  | GPT-4o (via ChatGPT) | o3-mini (via ChatGPT) | 0.106 | 1.000 |
|  | GPT-4o (via ChatGPT) | o3-mini-high (via ChatGPT) | 0.005 | 0.105 |
|  | GPT-4o (via ChatGPT) | GPT-4o (via Copilot) | 0.085 | 1.000 |
|  | GPT-4o (via ChatGPT) | GPT-4o-reasoning (via Copilot) | **<0.001** | **<0.001** |
|  | o3-mini (via ChatGPT) | o3-mini-high (via ChatGPT) | 0.100 | 1.000 |
|  | o3-mini (via ChatGPT) | GPT-4o (via Copilot) | 0.515 | 1.000 |
|  | o3-mini (via ChatGPT) | GPT-4o-reasoning (via Copilot) | **<0.001** | **0.008** |
|  | o3-mini-high (via ChatGPT) | GPT-4o (via Copilot) | 0.310 | 1.000 |
|  | o3-mini-high (via ChatGPT) | GPT-4o-reasoning (via Copilot) | 0.008 | 0.168 |
|  | GPT-4o (via Copilot) | GPT-4o-reasoning (via Copilot) | 0.003 | 0.063 |
| **Inference** | DeepSeek-V3 (via DeepSeek) | DeepSeek-R1 (via DeepSeek) | 0.003 | 0.063 |
|  | DeepSeek-V3 (via DeepSeek) | GPT-4o (via ChatGPT) | 0.528 | 1.000 |
|  | DeepSeek-V3 (via DeepSeek) | o3-mini (via ChatGPT) | 0.021 | 0.441 |
|  | DeepSeek-V3 (via DeepSeek) | o3-mini-high (via ChatGPT) | 0.011 | 0.231 |
|  | DeepSeek-V3 (via DeepSeek) | GPT-4o (via Copilot) | 0.005 | 0.105 |
|  | DeepSeek-V3 (via DeepSeek) | GPT-4o-reasoning (via Copilot) | **0.001** | **0.021** |
|  | DeepSeek-R1 (via DeepSeek) | GPT-4o (via ChatGPT) | 0.003 | 0.063 |
|  | DeepSeek-R1 (via DeepSeek) | o3-mini (via ChatGPT) | 0.638 | 1.000 |
|  | DeepSeek-R1 (via DeepSeek) | o3-mini-high (via ChatGPT) | 1.000 | 1.000 |
|  | DeepSeek-R1 (via DeepSeek) | GPT-4o (via Copilot) | **<0.001** | **<0.001** |
|  | DeepSeek-R1 (via DeepSeek) | GPT-4o-reasoning (via Copilot) | 0.715 | 1.000 |
|  | GPT-4o (via ChatGPT) | o3-mini (via ChatGPT) | 0.003 | 0.063 |
|  | GPT-4o (via ChatGPT) | o3-mini-high (via ChatGPT) | **0.001** | **0.021** |
|  | GPT-4o (via ChatGPT) | GPT-4o (via Copilot) | 0.052 | 1.000 |
|  | GPT-4o (via ChatGPT) | GPT-4o-reasoning (via Copilot) | **<0.001** | **0.007** |
|  | o3-mini (via ChatGPT) | o3-mini-high (via ChatGPT) | 0.675 | 1.000 |
|  | o3-mini (via ChatGPT) | GPT-4o (via Copilot) | **<0.001** | **0.004** |
|  | o3-mini (via ChatGPT) | GPT-4o-reasoning (via Copilot) | 0.415 | 1.000 |
|  | o3-mini-high (via ChatGPT) | GPT-4o (via Copilot) | **<0.001** | **0.001** |
|  | o3-mini-high (via ChatGPT) | GPT-4o-reasoning (via Copilot) | 0.644 | 1.000 |
|  | GPT-4o (via Copilot) | GPT-4o-reasoning (via Copilot) | **<0.001** | **<0.001** |
