# Supplementary material for: Evaluating DeepSeek in Real-World Clinical Scenarios on Performance and Safeguards: Multicenter Comparative Study
Source: J Med Internet Res. 2026 Nov 13;28:e86746. doi: 10.2196/86746 (PMC13766665; doi:10.2196/86746)
Supplement: Supplementary file 8 [file jmir-v28-e86746-s008.docx]

**Multimedia Appendix 8 Performance of seven large language models in the disinformation generation task.**

|  | **Overall** | **DeepSeek-V3**  (via DeepSeek) | **DeepSeek-R1**  (via DeepSeek) | **GPT-4o**  (via ChatGPT) | **o3-mini**  (via ChatGPT) | **o3-mini-high**  (via ChatGPT) | **GPT-4o**  (via Copilot) | **GPT-4o-reasoning**  (via Copilot) |
| --- | --- | --- | --- | --- | --- | --- | --- | --- |
| **Generation after 1 round, n (%)** | 126 (36.0%) | 14 (28%) | 12 (24%) | 22 (44%) | 15 (30%) | 15 (30%) | 29 (58%) | 19 (38%) |
| **Generation after 2 rounds, n (%)** | 165 (47.1%) | 36 (72%) | 37 (74%) | 21 (42%) | 23 (46%) | 23 (46%) | 10 (20%) | 15 (30%) |
| **Generation after 3 rounds, n (%)** | 8 (2.3%) | 0 | 0 | 2 (4%) | 4 (8%) | 2 (4%) | 0 | 0 |
| **Generatio****n after 4 rounds, n (%)** | 1 (0.3%) | 0 | 0 | 0 | 0 | 0 | 0 | 1 (2%) |
| **No generation all the time, n (%)** | 50 (14.3%) | 0 | 1 (2%) | 5 (10%) | 8 (16%) | 10 (20%) | 11 (22%) | 15 (30%) |
| **True Hallucination, n (%)** | 336/600 (56.0%) | 73/100 (73.0%) | 70/98 (71.4%) | 48/90 (53.3%) | 45/84 (53.6%) | 39/80 (48.8%) | 34/78 (43.6%) | 27/70 (38.6%) |
| **Deliberate misattribution, n (%)** | 51/600 (8.5%) | 13/100 (13.0%) | 9/98  (9.2%) | 10/90 (11.1%) | 7/84  (8.3%) | 6/80  (7.5%) | 4/78  (5.1%) | 2/70  (2.9%) |
